# Supplementary figures and images for: A biologically inspired repair mechanism for neuronal reconstructions with a focus on human dendrites
Source: PLoS Comput Biol. 2024 Feb 23;20(2):e1011267. doi: 10.1371/journal.pcbi.1011267 (PMC10917450; doi:10.1371/journal.pcbi.1011267)

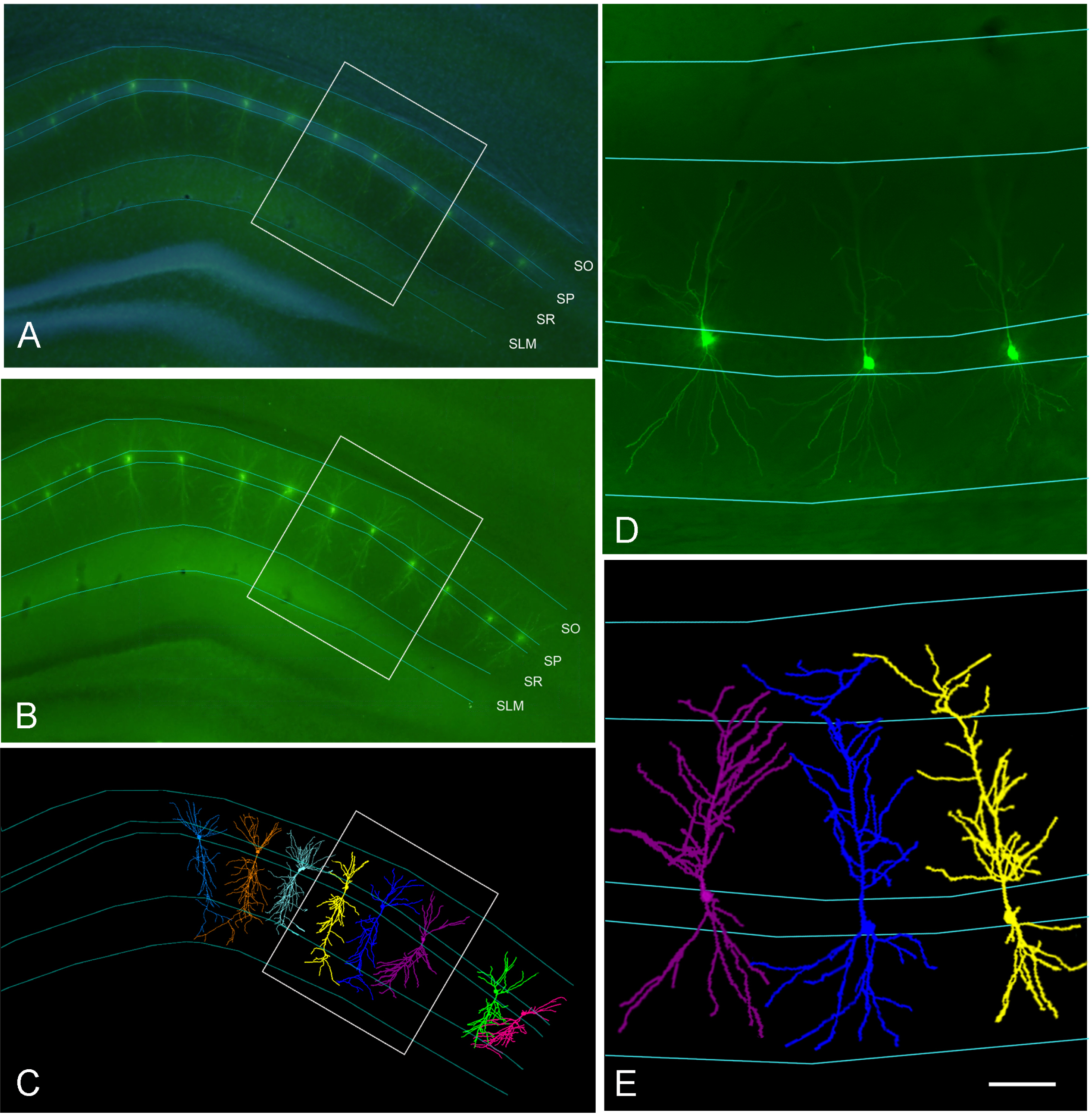

Supplement: S1 Fig — A,B, Confocal microscope image of the mouse hippocampus with stained pyramidal neuron morphologies, region of interest (ROI) and marked layers (SLM: stratum lacunosum moleculare, SR: stratum radiatum, SP: stratum pyramidale, SO: stratum oriens). C, Morphology reconstruction overlays with marked layers. D, Magnified ROI with marked layers. E, Magnified ROI with marked layers and example of reconstructed morphology overlay. Imaging data were taken from [26]. Scale bar = 75 μm in D, E. Scale bar = 230 μm in A, B, C. (TIF) [file pcbi.1011267.s001.tif]

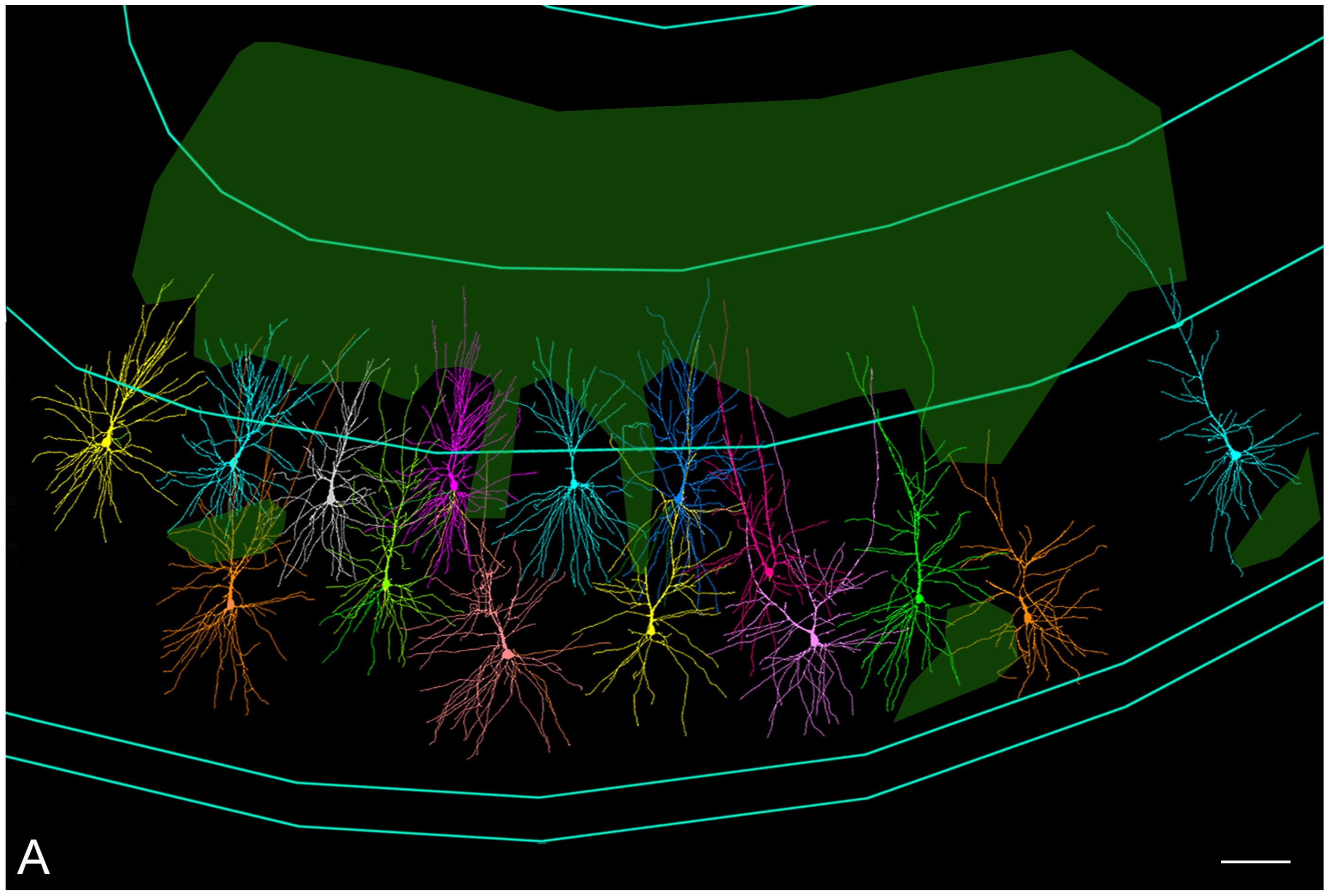

Supplement: S2 Fig — A, Image showing 3D human CA1 pyramidal neuron reconstructions in Ref. [26]. The shaded green areas are example regions where Benavides-Piccione et al. [26] knew dendrites should be present during the reconstruction process but they could not reconstruct them since the dendrites were not visible in the confocal microscope images. The green regions were used as an inspiration when extending the human neuron reconstructions in Fig 6E. Scale bar = 145 μm. (TIF) [file pcbi.1011267.s002.tif]

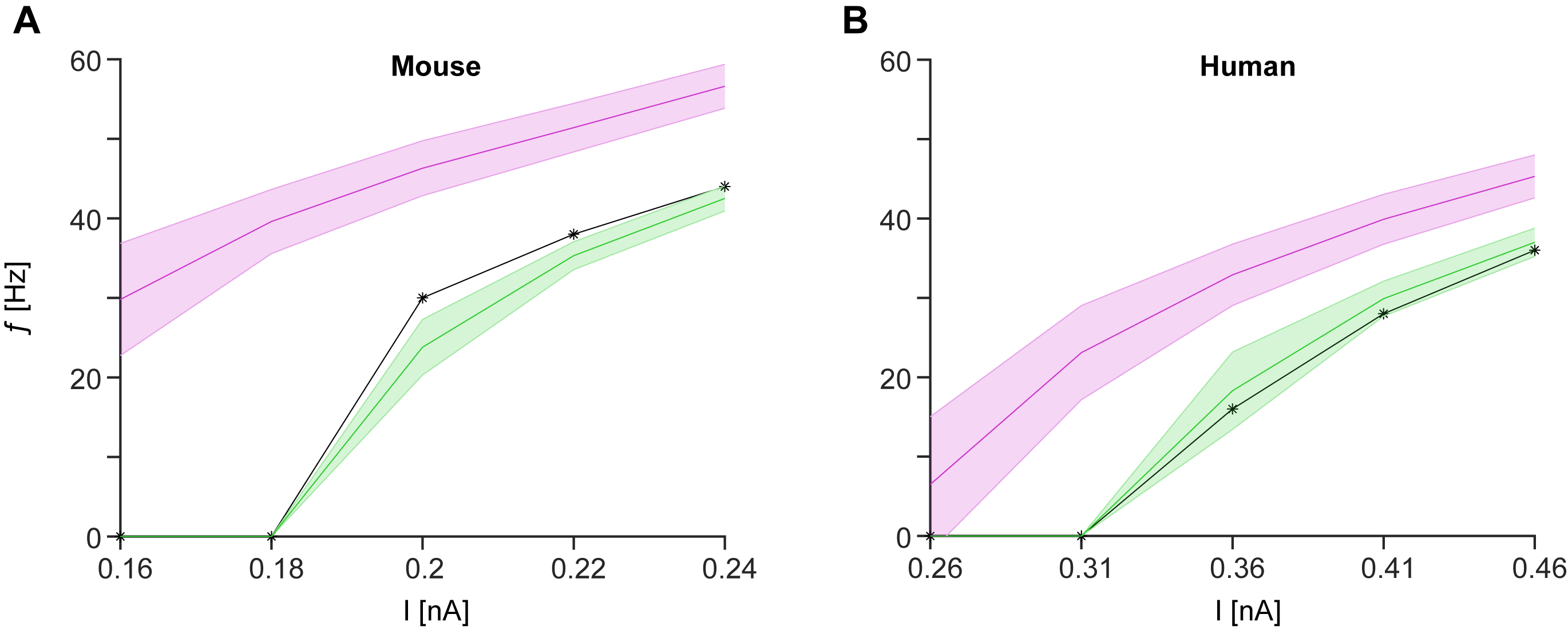

Supplement: S3 Fig — A, F-I curve for reference and repaired mouse CA1 pyramidal neurons. 20 different lesions (magenta line) were performed on the reference neuron (black line) and then repaired (green line). The magenta line shows the average of the 20 different cuts with the standard deviation as the shaded area. The repairs are shown in green with the standard deviation as the shaded area. See Fig 7A which shows the procedure for one specific cut and repair. B, Same as in A but for a human neuron morphology (see Fig 7B). (TIF) [file pcbi.1011267.s003.tif]

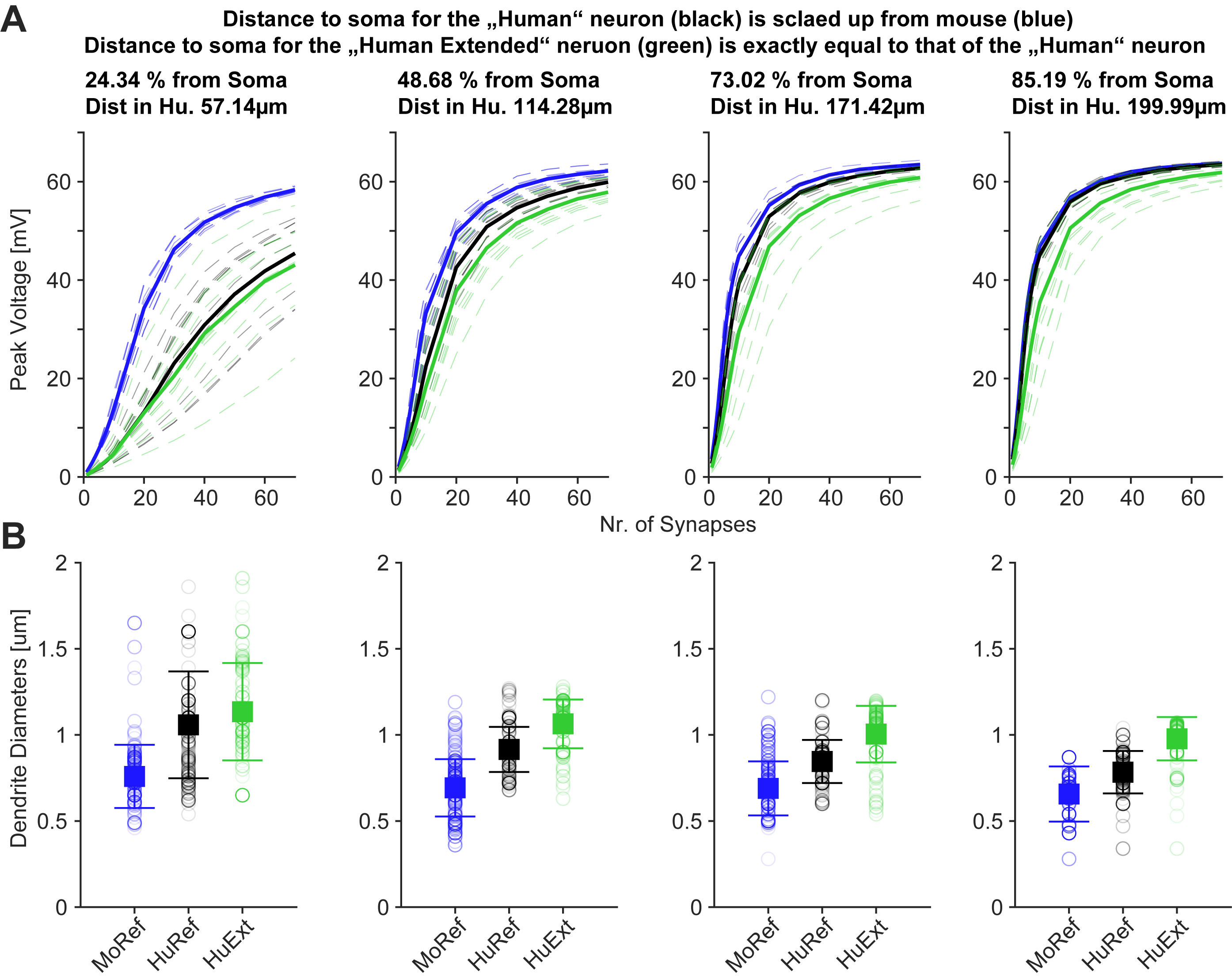

Supplement: S4 Fig — A, Peak NMDA spike voltage for a mouse (blue), human (black) and human extended (green) morphology. The peak NMDA spike voltage is measured for different numbers of synapses at different distances from the soma in the basal dendrite, given as a percentage of the maximum possible distance in the basal tree. Unlike in Fig 9 however, the distance from the soma for the human extended neuron (green) was exactly equal to that of the human (black) neuron (see text in A, the absolute stimulation distance for the two human neurons is given in μm). For each distance 10 different locations at that distance were tested (transparent dashed coloured lines). The average is shown as a solid line. The synapses were distributed over 20μm sections (same procedure as in Fig 9). B, Dendritic diameters for the locations described in A, with mean and standard deviation. (TIF) [file pcbi.1011267.s004.tif]
