# Supplementary material for: A biologically inspired repair mechanism for neuronal reconstructions with a focus on human dendrites
Source: PLoS Comput Biol. 2024 Feb 23;20(2):e1011267. doi: 10.1371/journal.pcbi.1011267 (PMC10917450; doi:10.1371/journal.pcbi.1011267)
Supplement: Attachment — Submitted filename: RebuttalFinal.pdf [file pcbi.1011267.s005.pdf]

JUSTUS-LIEBIG-UNIVERSITÄT GIESSEN

Bernstein Preis Computational Neuroscience Hermann Cuntz

Ernst Strüngmann Institute in Cooperation with Max Planck Society

FIAS Frankfurt Institute for Advanced Studies

To: **PLOS,**
1265 Battery Street, Suite 200,
San Francisco, CA 94111, USA

December 17, 2023

Dear editors of *PLoS Computational Biology*,

Thank you very much for your comments and suggestions regarding our manuscript entitled '**A biologically inspired repair mechanism for neuronal reconstructions with a focus on human dendrites**', which we had submitted to *PLOS Computational Biology*.

We are grateful to the reviewers for their suggestions to improve the manuscript. In particular reviewers asked for more in depth statistics to validate the results of the repair algorithm. This led to additional figures and explanatory text. Furthermore, we have revised the manuscript according to all the points raised by the reviewers. Please see a detailed point-by-point response below.

Please do not hesitate to contact us if you have further questions; we look forward to hearing from you,

Moritz Groden, Hannah M. Moessinger, Barbara Schaffran, Javier DeFelipe,
Ruth Benavides-Piccione, Hermann Cuntz, Peter Jedlicka

Dr. Moritz Groden
Justus-Liebig-Universität Gießen - Fachbereich Medizin
Institut für Medizinische Informatik, Rudolf-Buchheim-Str. 6, D-35392 Gießen

Responses to the comments of the editor and the reviewers

For the details of our revision please refer to the point-by-point answers to reviewer comments provided below.

Reviewer # 1

The authors test their morphology repair algorithm on human neurons and make a few predictions about the effects on electrophysiological properties. While the tool is useful and the aims are interesting, there is a general lack of sufficient quantitative details in the results, and some key analysis is missing.

Our response 1: Thank you, we have followed these suggestions by adding more quantitative detail to the results and extending the analysis. For example, we have added morphological statistics to Figure 3 (see below). We have also increased the number of repairs studied in both Figure 3 and 5, the latter of which (the old Figure 5) has been merged with the old Figure 6 (the merged new figure is now Figure 5). Furthermore, in line with reviewers' suggestions, we have added supplementary figures including effects on additional electrophysiological properties (see details below).

Major:

1. Fig2 D-F: authors should clarify what is the corresponding experimental profile, to assess the algorithm performance.

Our response 2: We have added a more detailed description of the experimental procedure in l. 134-138. "The authors of Song et al. 2012 state that class IV da neurons regenerate in an all-or-none fashion, meaning that a severed branch will either regrow or fail to do so entirely. The latter will leave the vacant area open to invasion by neighbouring branches. They report that in about 50% of branch injuries the severed stem would regenerate successfully, resulting in a bimodal distribution."

2. Authors should clarify the details of their algorithm visually and conceptually, and what bf means, and the implications of the distribution they plot.

Our response 3: Thank you for the suggestion. We have added a new panel A to Figure 2, where we clarify and showcase the impact of the bf (balancing factor) on a dendrite morphology when repairing a neuron. We have further introduced the details of morphological modelling in the following passages:
"Optimal wiring principles allow the dendritic structure to be described by locally optimised graphs, in which total length and path length are minimised (Cuntz et al., 2007; Wen and Chklovskii, 2008). An algorithm that weighs these two factors by a balancing factor bf can generate synthetic trees that reproduce biological dendrites (Cuntz et al., 2010, 2011). The impact of the balancing factor is showcased in Figure 2A by repairing an artificial 2D morphology using different values of bf. A small bf

(close to or equal to 0) favours minimising of total cable length, as opposed to the direct path length to the soma (or the signal travel time to the soma). In turn, short path lengths are favoured when bf is large (close to or equal to 1)." in l. 76-84.

3. Fig 3 should include metrics such as given in Fig2C to support claim of success. It's not clear why authors focus only on % regrowth from cut branch.

Our response 4: In line with the point of the reviewer, we have now added the same statistics/metrics in Fig. 3 as those in Figure 2. Additional descriptions were added:
"The granule cell repair was able to accurately match the reference number of branch points, while the total dendritic length and mean segment length were less reliably reproduced but still significantly improved over the cut version (Figure 3B, see RMSE as a percentage of the reference value). The Sholl distribution (Figure 3C) of the repair also showed a significant improvement over the cut neuron." in l. 183-187.
"To demonstrate that the algorithm works for any neuron type, the same procedure was applied to a mouse Purkinje cell (Chen et al., 2013) with many branches on the right side of Figure 3. Purkinje cells are known to minimise the material cost more than the conduction time, exhibiting a low bf , Figure 3E-H (same layout as for the granule cell). The morphological statistics were in good agreement with the reference, except for the mean segment length, which showed only a slight improvement as indicated by the RMSE. The histogram in Figure 3G shows the regeneration vs invasion statistics of the Purkinje neuron." in l. 192-199.

4. Fig 5 is rather qualitative, and should include metrics to compare the original/cut/repaired morphologies and support the claim of success. e.g. the metrics shown in fig 6, and/or distance between Sholl profiles, number of peaks etc. For example, bottom left reconstruction has one large second peak vs 3 small peaks in the original.

Our response 5: To address the concern of the reviewer, the number of different repaired neural morphologies is now reduced to three but the morphological statistics now include a total of 50 different neurons. Each of the 50 neurons has been cut and repaired similarly to the three example morphologies. We have merged the old Figures 5 and 6 into a new Figure 5, we have also added a comment stating the slight mismatches in the Sholl profiles in l. 239-243:
"The Sholl profiles showed a significant improvement over the cut dendrites, but did not exactly replicate the reference. An exact match was not possible due to the stochastic nature of the repair mechanism. For example, in Figure 5A (middle) the two right side peaks in the Sholl profile were present in the repair, but were more prominent compared to the reference."

5. Fig 5 top right, basal dendrites cut seem to be middle sections of the dendrites. Authors should clarify that case, and potentially the utility of their algorithm for such cuts.

Our response 6: We have removed this example from Figure 5 as it could be misleading. It was simply an arbitrary cut that happened to be in the middle section. Our algorithm is not specific to particular cuts and should be applicable to any cut position and orientation. In general, however, it becomes more difficult to produce an accurate repair when only minimal portions of the dendrite remain. This is because the algorithm analyses the remaining part of the dendrite to determine the growth parameters. We have added a section to the text that explains this in l. 252-254:

"The growth parameters for the algorithm are determined by analysing the remaining part of a cut dendrite, making repairs more difficult when only minimal dendritic material remains."

6. The results text lacks supporting quantitative analysis and statistics, e.g. corresponding to Fig 3 and Fig 6 – what is the correlation or RMSE between cut/original vs repaired/original, what is the effect size / statistical significance? Authors should use that to help the reader assess the lesser success for basal length per segment, for example.

Our response 7: We have added measures of the RMSE to Figures 2, 3 and the merged Figure 5 to quantify the size of the effects we are demonstrating. This should add supporting quantitative details. We have also added a section of text explaining where the algorithm was not as accurate as in other measures (l. 271-276):
"The statistical agreement is also indicated by the RMSE values, which were approximately one order of magnitude smaller for each measure except for the mean dendritic length per segment in the basal dendrite and for the mean diameter per segment in the basal dendrite. For the latter, even the values for the cut dendrites were a close match. The improvement of the repair was measurable but not as significant. For the former, the repair approximately halved the RMSE."

7. To support the success of the algorithm, in fig 6 Authors should plot performance on a larger data set than just 6 morphologies, to support statistical testing and claims, and also on different cell types.

Our response 8: To address this point of the reviewer, we have added 47 additional mouse CA1 pyramidal neuron cuts and repairs to the statistical analysis for a total of 50, which are now included in Figure 5. We have also added the statistics of 20 cuts and repairs for the different neuron cell types in Figure 3.

8. Fig 7 – should include quantitative aspects of the reconstruction. Even the qualitative nature of the reconstruction is unclear.

Our response 9: We assume here that the reviewer means the repaired (extended) human neuron morphologies rather than the original reconstructions by Benavides-Piccione et al. (2020). The published paper, Benavides-Piccione et al. (2020) provides detailed qualitative and quantitative descriptions of these reconstructions. As for the extended human neurons, we have now included a new supplementary Figure S2 that shows the target regions where dendrites are missing in the neuron reconstructions. Importantly, since this is a prediction, we do not have the ground truth in the form of 100% complete human reconstructions to assess the quantitative aspects of the repaired (extended) morphologies. The repaired morphologies are more complete after the in silico repair, as judged by the experts in human cellular neuroanatomy (R.B.-P. and J.d.F.), but we cannot use statistical measures to quantify this. We have added a statement regarding this in l. 289-294:
"Figure S2 shows examples of regions where dendrites were missing from the neuron reconstructions. The authors of Benavides-Piccione et al. (2020) knew that human CA1 pyramidal neuron dendrites were present in these regions, but could not reconstruct them because they were not visible in the microscopic images due to technical limitations and accidental lesions. We cannot evaluate the accuracy of the repair algorithm regarding the extended human neuron morphologies, as the model provides a prediction in this case."

9. Fig 8 – should include quantitative aspects – firing rate of the ref/cut/repaired cases, FI curve/gain, and the corresponding text should include stats and significance values. Also for 8C – what is the predicted change in rate or FI curve/gain?

Our response 10: We have added new F-I-curve inlays to Figure 8 and computed the discrete Fréchet distance from the reference model in all cases to compare the repaired neuron to the cut and reference and quantify the significance. We describe these new features as well as the predicted average gain for the extended neuron in new text sections:
"In the repaired neuron, the firing behaviour was restored as demonstrated by the F-I curves (Figure 7A, B insets). The firing rate of the cut neuron (magenta) was much higher than that of the reference (black) and repaired (green) neurons in both cases. The firing behaviour of the repaired neuron was close to that of the reference, although the firing rate was slightly lower. The discrete Fréchet distances between the cut-reference and repaired-reference electrophysiological curves show that the improvement of the repair was substantial (Mouse: cut-reference = 49.11; repaired-reference = 6.63; Human: cut-reference = 71.44; repaired-reference = 2). The Fréchet distance was approximately one order of magnitude smaller for the repair in both cases." in l. 303-311.
"The F-I curve inset underlines the reduction in excitability, predicting an average gain of $-16.67Hz$ for an extension depicted in the figure." in l. 327-328.

10. Fig 8 - authors should check the effect of cut and repair on other electrophysiological properties e.g. sag current, adaptation index.

Our response 11: The model by Jarsky et al. (2005) does not include HCN channels or slow K^+-channels that would produce a sag current or spike adaptation, respectively. Therefore, these measurements cannot be applied to the neuronal model presented in the old Figure 8. However, to investigate this issue, we have performed additional simulations in a model containing additional ion channels and have included the results in a new Figure 8 (the old Figure 8 is now Figure 7). We used the more detailed CA1 pyramidal neuron model by Poirazi et al. 2003a,b to analyse the effect of a repair on more detailed electrophysiological properties like the sag current, ISI, adaptation index etc. The new results as well as the new figure are described in a new text section in l. 329-358:
"To analyse the effect of repair on more detailed electrophysiological properties of a neuron, such as sag current, inter-spike interval (ISI) and adaptation index, we used a different model of CA1 pyramidal neurons from Poirazi et al. (2003a,b). The model in Jarsky et al. (2005) does not include HCN channels or slow K^+-channels, which would produce a sag current or spike adaptation, respectively. Therefore, such measurements can only be applied when using the model in Poirazi et al. (2003a,b), which was converted to T2N (Beining et al. 2017) in Cuntz et al. (2021) such that the morphology can be exchanged. Here, we used a mouse CA1 pyramidal neuron morphology from Šišková et al. (2014) as was previously done in Mittag, Mediavilla et al. (2023). The morphology was cut and subsequently repaired using our algorithm in Figure 8. The model in Poirazi et al. (2003a,b) became unstable for very large neurons such as those found in human tissue. The ion channel distribution in this model depends on the layers in CA1. Laminar regions were well defined within the model for mouse neuron morphologies but not for human neurons. For this reason, human neurons have not been included in Figure 8. The top of Figure 8 depicts example voltage traces from current clamp simulations of a repaired mouse CA1 pyramidal neuron for the reference (black), the cut (magenta) and the repair (green) using the model in Poirazi et al. (2003a,b) (a zoomed version is also depicted). The six plots below show different electrophysiological metrics for the three morphologies using the same colour scheme. In each case in Figure 8, we found that the repaired model (green line) was much closer to the reference (black line) than the cut model (magenta line), suggesting that repairing

the morphology restored the electrophysiological behaviour of the neuron. The cut neuron exhibited a reduced firing frequency but a larger negative voltage sag. For small stimuli, the firing frequency of the repaired neuron was almost perfectly matched to the reference, but began to deviate for larger stimuli. The inter spike interval (ISI) between the first and second spike as well as the spike adaptation index were all significantly increased for the cut neuron, with the half-width of the first spike being similar for all morphologies. Finally, the difference between the peak of the first and the second spike in the cut neuron was increased for small stimuli. For larger stimuli, the reference and repaired morphologies showed a steep increase, but the cut morphology did not. The spike adaptation index was larger for low stimuli in the reference and the repaired morphology, but decreased significantly for higher stimuli. In contrast, the adaptation index remained relatively constant for the cut morphology."

11. Fig 8 should examine the effect of different cuts as the authors did in fig 2, and plot the resulting metrics.

Our response 12: This is a valid concern. Therefore we have included a new supplementary Figure S3, where we check the effect different cuts and repairs have on the electrophysiological behaviour of the neuron. We found no significant impact on the firing behaviour for different cuts. We describe this matter in l. 311-320:
"To study the effect of different cuts and repairs on the electrophysiological behaviour we performed the same simulations as in Figure 7A, B but for different cuts on the same morphologies. The results are presented in Figure S3, where the reference F-I curve is plotted in black next to the average of 20 different repaired lesions in green with the standard deviation (cut morphologies in magenta). There was no notable difference in the recovery of firing behaviour for the different cuts, which is supported by the discrete Fréchet distances between the curves of the average repaired, reference and cut morphologies (Mouse: cut-reference = 55.32; repaired-reference = 6.92; Human: cut-reference = 24.28; repaired-reference = 3.15). The Fréchet distances for the average of the different repairs were similar to those shown in Figure 7A, B."

12. Fig 9 – authors should account for the cutting of the mouse morphologies as well, for a proper comparison of repaired prediction between species. Also, authors should highlight what the prediction from C is. The relevance of part D is unclear and should be either be used in the context of the NMDA analysis or removed from the figure.

Our response 13: Figure 9 attempts to replicate the difference in synaptic thresholds for NMDA spike generation between mouse and human found by Testa-Silva et al. (2022). It shows that the incomplete human morphology can only maintain its higher threshold compared to the mouse close to the soma, whereas the extended human morphology exhibits a higher threshold also further away from the soma. This shows that truncated or incomplete human morphologies confound simulations of dendritic spikes. Our prediction stands since the figure demonstrates the importance of dendrite repair for more realistic simulations of human dendritic properties. We know from the findings of Testa-Silva et al. (2022) that the human neuron should exhibit a higher threshold than the mouse neuron, which it only does consistently with a dendrite extension/repair. We have added text in l. 389-394 to make this clearer:
"Therefore, an incomplete human neuron (incomplete due to reconstruction limitations) did not exhibit a different NMDA threshold on the outer parts of the dendrites. Completing the neuron with an extension using our algorithm restored this behaviour, increasing the threshold and therefore resulting in a lower peak voltage (Figure 9C Right). Thus the extended human neuron reproduced the

findings of Testa-Silva et al. (2022) more accurately than the incomplete human reconstruction." We would like to argue that the figure would not gain more information by including a cut mouse morphology since we have no data to validate the cut morphology against. We have included the diameter measurements at the different stimulation sites to closely replicate the procedure and findings of Testa-Silva et al. (2022) and to provide a mechanistic intuition for the lower dendritic spike threshold in thick human dendrites as compared to thin mouse dendrites. Consistent with our simulations, in their paper, Testa-Silva et al. (2022) detect a difference in dendritic diameter at the stimulation sites for the two species. They also provide evidence that the larger human dendritic diameters are a major cause for the higher NMDA threshold. We have added text to make this clearer in l. 411-413: "The differences in diameter were also found by Testa-Silva et al. (2022), who provide evidence that the higher NMDA spike threshold in human neurons is likely due to larger dendritic diameters."

13. Fig 9 - the repaired human morphology has a different % from soma location, how does that affect the analysis? Authors should analyse and plot results for absolute distance comparison as well, to address this confound.

Our response 14: Thank you for your suggestion. We have repeated the same simulation as in Figure 9, where the absolute distances for the human and human extended neuron are exactly the same. The plots have been added as a Supplementary Figure S4. We found no significant impact on the results, probably due to the differences in stimulation site distance in the original experiment being less than $20\mu m$, which is within the size range of a segment where synapses were distributed. We have added text in l. 394-404 explaining these results.
"Since the human extended neurons (green) were even larger than the incomplete human neurons (black), the absolute distance of the stimulation site from the soma was not the same in these two cases. To investigate whether this discrepancy in distance had a significant impact on the analysis presented in Figure 9, we re-ran the same simulation as in Figure 9, where the absolute stimulation distances from the soma were the same in both human and human extended neurons (Figure S4). The results in Figure S4 suggest that the difference in stimulation distance between the two human neurons had no significant effect on the NMDA spiking behaviour, as the results were similar to Figure 9. As the difference in stimulation distance in Figure 9 was only $\sim 10 - 20\mu m$, which was in the range of the size of the sections in which synapses were distributed, no significant effect was to be expected."

14. Authors should illustrate and discuss the limitations of their algorithm. Are there cases where it struggles?

Our response 15: We have added a paragraph detailing the shortcomings of our algorithm:
"Our algorithm is unlikely to be suitable for repairing astrocytes or other glial cells, as it has not been validated for growing them specifically. It is currently unclear whether glial cells follow similar growth rules to neurons, but a recent study published a 3D editing tool for glial cells (Keto and Manninen, 2023) to facilitate future detailed simulations of glial cells. If future research can confirm that MST_tree is indeed suitable for glial cell repair, the fix_tree function would need to be re-evaluated in this regard. Alternative growth algorithms may be incorporated into the fix_tree function in the future." in l. 551-557.
"It is important to note that the repairs made by our algorithm are not perfect. Visually, they do not always resemble their biological counterparts exactly, as can be seen in Figure 2A, B and Figure 3E. These examples are morphologies with low bf. In terms of the morphological statistics the algorithm was not able to perfectly replicate the Sholl profile of the reference neuron (Figure 5A).

This mismatch was, at least in part, due to the stochastic nature of the algorithm. Nevertheless, the match was close, but in the most distal parts of the dendrites, the number of branches was slightly too high. In addition, the volume occupied by the repaired dendrites was slightly smaller compared to the reference morphology. Due to these discrepancies we find that we were able to restore the electrophysiological behaviour well, but the gain in the F-I curve of the repair did not match the reference exactly (Figure 7A, B, C inlays). As for the more detailed electrophysiological properties the repair also represents a close match to the reference (Figure 8). The growth algorithms can be further refined in the future, e.g. based on developmental data (Ferreira Castro et al., 2020; Baltruschat et al., 2020)." in l. 564-576.

15. Authors should clarify the dendritic ion channels and their distributions, are they a function of the relative or absolute distance from soma, and how does the repair affect the distributions?

Our response 16: There is one K^+ conductance that increases linearly as a function of the direct path length to the soma (absolute distance from the soma along the dendrite). We have described this in the "Methods Electrophysiology (*T2N*)" part of the text. We have added text in l. 721-724 explaining how the repair affects this distribution:
"As the repair extended the cut dendrites, we stretched the ion channel distributions along the newly formed dendrites accordingly. In addition, in new simulations (Figure 8), we have used an active model from Poirazi et al. (2003a,b) that contains a set of active channels which are described in more detail in the next paragraph."

Minor:

1. Number of figures can be reduced by combining e.g. fig5+6 (with fewer than 6 examples if necessary).

Our response 17: Thank you for the suggestion. Done.

2. Fig 2e – for better clarity, authors should instead plot length of cut on x axis and something like a violin plot of the distribution of % growth from cut.

Our response 18: We followed the suggestion of the reviewer and reversed the axis of the plot and added normalised binned histograms to the original data points to make the results easier to understand.

3. Fig 6 caption should clarify what measure they refer to by diameter per segment – is it average?

Our response 19: It is the average diameter per segment. We have updated the figure description to explain the measures in more detail.

4. Fig 9 should use a different colour for mouse, to avoid confusion with previous figures.

Our response 20: Thank you, we have changed the colour to light blue for better clarity.

5. Authors should clarify if users can use the tool in other languages such as python, and what file types are supported.

Our response 21: We have added a statement to the first paragraph of the "The fix_tree function of the repair algorithm" section of the "Materials and methods" chapter. It now states that the algorithm is currently only available in MATLAB and what the supported file types are.

Reviewer # 2

Reviewer comments: The manuscript presents an algorithm provided in TREES Toolbox to repair incomplete dendritic reconstructions either by regenerating from the cut end of the branch or invading from adjacent branches. This algorithm and provided graphical user interface are highly needed in the field, especially related to human cells. The authors have used the algorithm with both mouse and human neuron morphologies. They have used the algorithm with whole neuron morphologies by cutting a part of the morphology away and then repairing it, and finally comparing the original (reference) morphology to the cut and repaired ones. They have also repaired morphologies that never were whole neuron morphologies. Electrophysiological behavior was also simulated with the reference, cut, and repaired morphologies, but more detailed analysis is needed to understand in detail how similar the membrane potentials are in these different cases. Figure 9 shows interesting results when comparing mouse, human, and human extended morphologies. After testing the fix_tree_UI a little bit, the algorithm is working but I could have received better results with better instructions. A video file and a manual showing how to run and use the tool would be helpful for the users.

Our response 22: Thank you. We have addressed the request for a more detailed electrophysiological analysis and have therefore provided additional results as well as new supplementary figures (see details below). We have also created a tutorial video and will provide it as an upload to the YouTube channel of the TREES toolbox.

Detailed comments:

1) Provide in the website of TREES Toolbox a video file and manual how to use fix_tree_UI for easy use of the tool. Provide information and figures in the manual how the target points should be chosen because it took long time to get realistic-looking dendrites. In addition, on l. 88: Please, be more specific by explaining what you mean by "target points".

Our response 23: We have followed the suggestion of the reviewer and created a video and a brief manual with an explanation of target point distribution (URL: `https://www.youtube.com/watch?v=hilJ06l_IgM`). The explanation of "target points" was also added to the text on l. 84-88: "Once target points (target points are successively connected to the dendritic tree according to optimal wiring principles weighted by bf) are distributed within a cell-type specific dendritic density

field, they can be connected to a tree structure according to these optimised wiring costs in e.g. fly (Cuntz et al., 2008) or mouse (Cuntz, 2012) as well as in some axons (Budd et al., 2010)."

2) Please, test the tool for astrocytes and possibly also for other glial cell types. At least discuss about it in discussion and if the tool is succeeding with glial cells, please add a figure about the morphology regeneration and invasion, testing the functionality is not needed.

Our response 24: The `MST_tree` function from Cuntz et al. (2010) has not yet been validated to generate artificial glial cells. It is still unclear whether glial cells follow similar wiring constraints as neurons. Therefore it is impossible for us to make that claim and will require starting a new project with glial datasets. However, as suggested by the reviewer, we have added a statement in the discussion in l. 551-557 to mention this possibility as an outlook:
See Reviewer # 1, response 15.

3) Consider discussing how your tool is different to other tools that are not cited in the manuscript, such as NETMORPH and CX3D. Furthermore, consider adding references related to dendritic growth models, such as Kirchner et al. (Dendritic growth and synaptic organisation from activity-independent cues and local activity-dependent plasticity).

Our response 25: Thank you for the suggestion. We have added a paragraph in l. 606-615 where we discuss the relationship of these models with our repair algorithm approach:
"Our tool does however, not consider the interactions between different neurons during growth such as other morphological models such as CX3D (Acimovic et al., 2011) and one in Kirchner et al. (2023). Kirchner et al. (2023) use an activity-driven algorithm where neuronal growth is determined by the activity of nearby potential synapses. The approach of CX3D focuses on chemical gradients and mechanical forces that can generate layer-specific branching patterns. A similar morphological model, NETMORPH, by Koene et al. (2009), grows neurons based on a stochastic branching outgrowth mechanism that does not use any extracellular cues. Modelling and completing multiple neuron types is likely to be more difficult using these alternative approaches, as the neuronal branching patterns in these models depend on many parameters."

4) In many occasions, you use "cell type" when actually "neuron type" would be more appropriate. Consider changing this to sentences that are not specific enough. You often write "human morphology" and "mouse morphology", but please be more specific and use "human neuron morphology" and "mouse neuron morphology".

Our response 26: Thanks. It is indeed more accurate to refer to neurons rather than just cell types and morphologies. We have changed this throughout the text.

5) Please, add Data availability statement.

Our response 27: We have added a Data availability statement.

6) Figure text of Figure 2: Explain more clearly in A and B, Left, that they show the branches in magenta that will be severed: e.g., "Reference Drosophila larva Class IV morphology in which the branches that will be severed deliberately are marked in magenta." Write "B, Right, Sample" instead of "B, Sample".

Our response 28: We have updated the figure description.

7) Figure 3: It seems that the bars are not adding up to 500, so please make sure the y-axis is correct.

Our response 29: Thank you for pointing this out! There was a bug that did not show the entire length of the y-axis. The issue has been fixed and the bars add up to 500 now.

8) Figure text of Figure 3: Explain in Methods clearly and also shortly in Results or in this figure text what is the difference between fix_tree and MST_tree. Explain here if one or both are used in A-C.

Our response 30: We have added text explaining the difference between *fix_tree* and *MST_tree* in both Results and Methods (l. 672-678). *fix_tree* always calls *MST_tree* so both are used in all cases: "The fix_tree function analyses the input neuron to set the growth parameters. It also distributes the target points in the growth volume, and identifies the incomplete (cut) terminals of the input neuron to restrict growth to those locations if needed. fix_tree then calls the MST_tree function, iterating with different numbers of target points until the number of branch points matches the desired value as closely as possible. fix_tree then edits the output tree, applying a jitter, adjusting diameters, and more."

9) Figure text of Figure 4: Please, be more specific where items 1-2 showing on top of the figure are done because those are not provided in the GUI. Specify also that extra tools (inpolyhedron) are needed for this. Write "Graphical-User-Interface" as "Graphical User Interface".

Our response 31: Thank you for your comment. We have updated the figure text and added a statement in l. 642-645 clarifying that the algorithm requires the *inpolyhedron* function in MATLAB: "The `fix_tree` algorithm and UI are, like the *TREES toolbox*, are only available in MATLAB (`fix_tree` and `fix_tree_UI` require the `inpolyhedron` function in MATLAB in order to operate. This was also explicitly mentioned in the YouTube tutorial)."

10) Figure text of Figure 6: To make the figure more clear, please explain the abbreviations used in the titles of the sub figures. Thus write open the titles as you have done in the results section: Total number of branch points, total dendritic length, dendritic length per segment and the diameter per segment for apical and basal arbours.

Our response 32: We have updated the figure text which is now part of the new Figure 5.

11) Figure 8: Please add more analysis of the membrane potentials so that the reader can see in detail how similar the membrane potentials are in reference, cut, and repaired morphologies: e.g., ISI, frequency of spikes, peak value, etc. Only after these results, the possible similarity can be stated.

Our response 33: We have added F-I curve inlays to Figure 8 (now Figure 7) as well as a new supplementary Figure S3 evaluating the impact of different cuts and repairs on the firing behaviour of the neurons. The discrete Fréchet distances between the reference, cut and repair show a significant improvement of the repaired neuron. Additionally we have added a new Figure 8 where we analyse the effect of a neuron repair on more detailed electrophysiological properties such as the ISI, frequency of spikes, spike adaptation index, first spike half width, difference in peak value between spike 1 and 2 and the sag voltage. We use the compartmental model by Poirazi et al. (2003a,b) since these measures would be impossible to quantify using the model by Jarsky et al. (2005) as it lacks the neccessary ion channels. The text describes these new features in the following sections:
"In the repaired neuron, the firing behaviour was restored as demonstrated by the F-I curves (Figure 7A, B insets). The firing rate of the cut neuron (magenta) was much higher than that of the reference (black) and repaired (green) neurons in both cases. The firing behaviour of the repaired neuron was close to that of the reference, although the firing rate was slightly lower. The discrete Fréchet distances between the cut-reference and repaired-reference electrophysiological curves show that the improvement of the repair was substantial (Mouse: cut-reference = 49.11; repaired-reference = 6.63; Human: cut-reference = 71.44; repaired-reference = 2). The Fréchet distance was approximately one order of magnitude smaller for the repair in both cases. To study the effect of different cuts and repairs on the electrophysiological behaviour we performed the same simulations as in Figure 7A, B but for different cuts on the same morphologies. The results are presented in Figure S3, where the reference F-I curve is plotted in black next to the average of 20 different repaired lesions in green with the standard deviation (cut morphologies in magenta). There was no notable difference in the recovery of firing behaviour for the different cuts, which is supported by the discrete Fréchet distances between the curves of the average repaired, reference and cut morphologies (Mouse: cut-reference = 55.32; repaired-reference = 6.92; Human: cut-reference = 24.28; repaired-reference = 3.15). The Fréchet distances for the average of the different repairs were similar to those shown in Figure 7A, B." in l. 303-320.
See also Reviewer # 1, response 11 (manuscript l. 329-358).

12) Figure text of Figure 9: Explain in the figure text, if or if not the human morphology presented is the same human morphology that is extended. Add a comma before "respectively". "μm" should not be in cursive. Is "purple" actually "magenta"?

Our response 34: We have updated the figure text to state that the human neuron morphology presented is indeed the one that is being extended. We have changed the purple colour to light blue as it was not supposed to be magenta. This is because the now blue dendrites are not cut in any way and cuts are indicated by magenta in all other cases.

13) You could cite your figures more in Discussion.

Our response 35: We have added figure citations in the Discussion where appropriate.

14) The order of references in the sentences is not always following any rules, e.g. oldest articles first/alphabetical order. Please, note that PLOS Comput Biol uses numeric citation style.

Our response 36: We have adjusted the order of cited articles so that the oldest comes first and now

we use the PLOS citation style.

15) l. 130-136: This paragraph could be reworded in such a way that the readers understand that the tool is working properly. The first sentence can give an impression that the invasion was undesirable. Furthermore, explain better that Fig. 2A, Left and 2B, Left show the original reference morphology where branches that will be severed are shown in magenta.

Our response 37: We have added a more detailed descriptions of both the figure and the fact that both modes of regrowth are desirable and therefore the algorithm is working properly in l. 126-129: "The model, just like the experiments showed two different possibilities for regrowth. Sometimes the synthetic regrowth invaded the available space, with new branches emerging from adjacent branches (Figure 2B, the branches in magenta on the left were intentionally severed and then repaired, as shown by the synthetic branches in green on the right)."

16) l. 160: You have explained the balancing factor (bf) also on l. 152-154. Maybe it would be more clear to define it well the first time you use it in Results, thus in l. 152-154.

Our response 38: Thank you for the suggestion. We have added a new panel to Figure 2 with a detailed explanation of the balancing factor. The bf is further explained in the figure text as well as in l. 80-84:
"The impact of the balancing factor is showcased in Figure 2A by repairing an artificial 2D morphology using different values of bf. A small bf (close to or equal to 0) favours minimising of total cable length, as opposed to the direct path length to the soma (or the signal travel time to the soma). In turn, short path lengths are favoured when bf is large (close to or equal to 1)."

17) l. 193-194: Specify in more detail what you mean by "arbitrary morphologies". Are other cells than neurons also possible?

Our response 39: We have changed this statement to "arbitrary neuronal morphologies" to make it more clear.

18) l. 426: Please, use "Intellectual disability" instead of "mental retardation".

Our response 40: Adjusted, sorry about this.

19) l. 431: Add a reference to "Allen Brain Atlas Data Portal".

Our response 41: We have added the URL of the Allen Brain Atlas Data Portal:
"Allen Brain Atlas Data Portal (`https://portal.brain-map.org`)." in l. 525-526.

20) l. 561: Please be more specific that the user has to run fix_tree_UI in MATLAB to use the tool and not fix_tree.

Our response 42: We have added text specifying that *fix_tree_UI* needs to be executed (l. 699-700): "The GUI can be accessed by running `fix_tree_UI` which uses the `fix_tree` function."

Smaller typos:

21) There are some grammatical mistakes in the GUI in the repair panel in the descriptions of the top two items (e.g. engabled).

Our response 43: Thank you! The descriptions have been updated.

22) Sometimes you write "TREES Toolbox" and sometimes "TREES toolbox". Same for "arbor" and "arbour".

Our response 44: The terms have been standardised.

23) l. 121: Add comma after "Branches".

Our response 45: Comma has been added.

24) Figures 2C and 4, write "Rep." as "Repaired".

Our response 46: Has been adjusted in Figures 2, 3 and 4.

25) l. 149: "mm" should not be in cursive. l. 271-272: "nA" and "ms" should not be in cursive. l. 294, 500, 587, 599, 602, 605: "µm" should not be in cursive. l. 571: "ms" should not be in cursive. l. 604: "pS" should not be in cursive.

Our response 47: Font styles have been updated.

26) l. 177: Write "has was cut" as "was cut".

Our response 48: Done.

27) Figure text of Figure 7: Write "intracellularly injected pyramidal cells" as "stained pyramidal cells". Write "B" as "B,".

Our response 49: Done.

28) l. 302, 308, 317: Is "purple" actually "magenta"?

Our response 50: We have changed the purple colour to blue since it was indeed misleading. Light blue was chosen on purpose to signify that these dendrites were not cut.

29) l. 335-336: Add a comma before and after: "i.e. path length to the root (Cuntz et al., 2010)".

Our response 51: Done.

30) l. 403: Write "al. (2021)" as "al., 2021".

Our response 52: Done.

31) l. 462: Write "such as Abdellah et al. (2018)" as "such as NeuroMorphoVis (Abdellah et al., 2018)".

Our response 53: Done.

32) Last paragraph on page 33: "μm" should not be in cursive.

Our response 54: Done.

33) l. 581: Write "channel" as "channels".

Our response 55: Done.

34) l. 588: Add empty space between paragraphs.

Our response 56: Done.

35) l. 594-595: Write "euclidean" as "Euclidean".

Our response 57: Done.

36) l. 603: Should "length" be "in length"?

Our response 58: Done.

37) Figure text of Figure S1: Make "E" bold and not as cursive.

Our response 59: Done.

Reviewer # 3

In Groden et al., inspired by biological regrowth in severed Drosophila dendrites, the authors propose that such approach could be deployed to repair incompletely reconstructed dendritic morphologies. The authors claim that their repair algorithm successfully recovered the incomplete parts of the dendritic trees and therefore could be used to simulate and predict their electrophysiological responsiveness. The algorithm itself is not novel as has been previously used and published to generate morphologies but is used and suggested for the first time to repair incompletely reconstructed morphologies. As I have pointed out below in my detailed comments that some other algorithms do exist which are deployed by Blue Brain Project team in repairing dendritic morphologies, the text in the manuscript does not capture it as it and claim that their algorithm is first one to repair the missing dendrites. Whether the existing algorithms do good job or not can be debated but should be mentioned for what those algorithms are meant to do. Besides that, the authors should improve some statistical analysis of morphological features to make effectiveness of their repair algorithm more outstanding.

Our response 60: Thank you for your constructive comments. It was never our intention to claim that our repair algorithm is the only existing one. We agree that we need to do a better job detailing the achievements of previous algorithms that came before ours. In terms of improving the statistical analysis of our algorithm, we have added more data and statistics to Figures 2, 3, 5 and 7 (Figure 5 is now 5 and 6 combined). Furthermore we have included a new Figure 8 that provides more in depth analysis of electrophysiological properties of repairs, as well as three supplementary figures that add details to Figures 6, 7 and 9.

Detailed Comments:

1. Lack of objective measure to support the claim that the morphology has been repaired successfully. Although providing evidence for bimodal regrowth generated by the repair algorithm is interesting, i am not convinced if it is essential to retrieve the functional properties of the neurons. Therefore better statistical comparison must be performed to compare uncut and repaired morphologies. e.g. when i see Purkinje cells in Fig 3B, the repaired one looks very different from the original one, so what makes the authors to claim that the repair algorithm worked? In Fig 3A, all the dendrites are reaching towards the top whereas the repaired ones are terminating far from the boundary. I understand that it might be difficult to establish the ground truth for repairing morphologies but I am sure its possible to compare multiple morphological features with statistical tests to report some objective measurements. The authors have tried to present morphological measurements in Fig.5 and 6. But in my opinion these statistics are not sufficient to claim successful repair. I clearly see higher branchings in Sholl distribution of first two repaired morphologies as compared to the original ones in Fig 5. Surprisingly, I don't see that in Fig 6, so I am not sure how to explain higher branch crossings in Sholl analysis for apical tuft.

Our response 61: We have added statistical analyses to Figure 3 detailing the nature of the repairs. We have also added more data to Figure 5 to make a better point when stating the success of the

repair. We agree that the repairs do not present a perfect match of the reference in all statistical measurements. To provide better evidence of how closely our repairs match the reference we have included the RMSEs as a percentage of the reference value. The Sholl profiles are indeed a close but not a perfect match. To state the shortcomings of our algorithm we included new text sections:
"The granule cell repair was able to accurately match the reference number of branch points, while the total dendritic length and mean segment length were less reliably reproduced but still significantly improved over the cut version (Figure 3B, see RMSE as a percentage of the reference value). The Sholl distribution (Figure 3C) of the repair also showed a significant improvement over the cut neuron." in l. 183-187.
"The morphological statistics were in good agreement with the reference, except for the mean segment length, which showed only a slight improvement, as indicated by the RMSE." in l. 196-197.
See also Reviewer # 1, response 5 (manuscript l. 239-243).
"The statistical agreement is also indicated by the RMSE values, which were approximately one order of magnitude smaller for each measure except for the mean dendritic length per segment in the basal dendrite and for the mean diameter per segment in the basal dendrite. For the latter, even the values for the cut dendrites were a close match. The improvement of the repair was measurable but not as significant. For the former, the repair approximately halved the RMSE." in l. 272-277.
See also Reviewer # 1, response 11 (manuscript l. 564-576).

2. the dendritic repair algorithm presented in Anwar et al 2009 does aim to add new branches to existing dendritic trees. How thoroughly they validated their algorithm can be debated but such a tool already existed. Please clarify this in the top paragraph (L70-80) on page 7. Same in Discussion "Relationship to other morphological models" L460-469.

Our response 62: Thank you for pointing this out. We had already cited the work but now have included text detailing the model by Anwar et al. 2009 in l. 583-588:
"Anwar et al. (2009); Coste et al. (2021) have presented a neuron repair tool in *NeuroR*. Their algorithm focuses on growing from severed ends only and has been validated on a single layer 2 pyramidal neuron. Importantly, such repair algorithms should restore the dendritic morphology as well as the original electrophysiological behaviour of a neuron in order to improve scientific inferences about neuronal functionality based on the repaired data. To date, there has been no electrophysiological validation of morphological repair algorithms."

3. Comparison is Fig 2C is not clear to me especially for Nr of branches and Total dendritic length. I would suggest running statistics (between Cut and Rep groups) and report p-values to see if those two classes are significantly different from each other or not.

Our response 63: Thank you for the suggestion. We have included the RMSE between the reference and the cut and repaired neurons as a percentage of the reference value respectively to give a better indication of the success of the repair.

4. It is very interesting and reasonable approach to test repair algorithms on reconstructions where complete data is available and extend the approach to the reconstructions where complete data is not available but research scientists must use caution when making claims using such approach and the readers should be warned of the limitations in clear words. Extending this approach to repair incompletely reconstructed human neurons and simulating those

neurons to provide predictions is a bold claim. As shown by the authors, the electrophysiological properties of these neurons are usually tested by current clamp recordings often at soma or occasionally at proximal apical dendrites, so matching the responsiveness at soma or proximal dendrites does not ensure matching characteristics at distal dendritic levels. I understand that these approaches are commonly used in neuroscience community. Authors should mention all these limitations so that the claim of successful repair does not look inflated.

Our response 64: A very important point. We have added text in both the Results and Discussion sections, mentioning the potential flaws and shortcomings of our algorithm:
"The granule cell repair was able to accurately match the reference number of branch points, while the total dendritic length and mean segment length were less reliably reproduced, but still significantly improved over the cut version (Figure 3B, see RMSE as a percentage of the reference value). The Sholl distribution (Figure 3C) of the repair also showed a significant improvement over the cut neuron." in l. 183-187.
"The morphological statistics were in good agreement with the reference, except for the mean segment length, which showed only a slight improvement as indicated by the RMSE. The histogram in Figure 3G shows the regeneration vs invasion statistics of the Purkinje neuron." in l. 196-199.
See also Reviewer # 1, response 5 (manuscript l. 239-243).
See also Reviewer # 1, response 7 (manuscript l. 272-277).
See also Reviewer # 1, response 15 (manuscript l. 551-557).
See also Reviewer # 1, response 15 (manuscript l. 564-576).
We also state that extending a neuron properly using the repair algorithm requires expertise from the user, as they have to have detailed knowledge of where dendrites are missing. This is emphasised by the new supplementary Figure S2, that marks out areas in the human CA1 region where dendritic material could not be reconstructed: "While this approach is highly flexible and gives the user complete freedom to choose where to grow the morphology, it does place an emphasis on the user's experience, anatomical knowledge and intuition. As shown in Figure S2, the user needs to have in-depth knowledge of where to grow the missing dendritic material. In all cases, repaired dendrites should be considered as a model prediction that is useful for improving incomplete reconstructions but requires further experimental testing." in l. 532-537.
